# Wolcott-Rallison Syndrome with Novel EIF2AK3 Gene Mutation

**DOI:** 10.4274/jcrpe.3065

**Published:** 2016-12-01

**Authors:** Fatih Gürbüz, Bilgin Yüksel, Ali Kemal Topaloğlu

**Affiliations:** 1 Ankara Pediatric Hematology-Oncology Training and Research Hospital, Clinic of Pediatric Endocrinology, Ankara, Turkey; 2 Çukurova University Faculty of Medicine, Department of Pediatric Endocrinology, Adana, Turkey

**Keywords:** Wolcott-Rallison syndrome, neonatal diabetes, epiphyseal dysplasia, EIF2AK3

## Dear Editor,

Wolcott-Rallison syndrome (WRS; Online Mendelian Inheritance in Man 226980) is an autosomal recessively inherited disorder characterized by neonatal insulin-dependent diabetes mellitus, skeletal dysplasia (epiphyseal dysplasia), acute hepatic and/or renal dysfunction, exocrine pancreatic insufficiency, neutropenia, developmental delay, and growth retardation (1,2). This syndrome is caused by mutations in the gene encoding eukaryotic translation initiation factor 2a kinase 3 (EIF2AK3), and to date, more than 60 cases have been reported (2,3).

A female Kurdish infant at 4 months of age had been diagnosed to have neonatal diabetes when admitted with an episode of diabetic ketoacidosis. Her parents were first-degree cousins. At diagnosis, laboratory findings (reference ranges) were as follows: glucose 492 mg/dL (70-105), C-peptide 0.001 ng/mL (0.9-4.3), insulin 0.2 µIU/mL (1.9-23), and HbA1c 15.2% (4.8-6.0). Her liver enzymes [aspartate aminotransferase (AST), alanine aminotransferase (ALT)], thyroid stimulating hormone, thyroxine, blood urea nitrogen, and creatinine levels, and neutrophil count were in normal ranges. Type 1 diabetes-associated autoantibodies (islet cell antibody and glutamic acid decarboxylase antibody) were negative.

At the age of eight months, the patient was admitted because of acute hepatic failure (on treatment with a regimen of insulin detemir and insulin lispro injected three times a day). ALT and AST levels (822 U/L and 1559 U/L, respectively) were elevated with no hepatomegaly. Viral hepatitis markers were negative. Additionally, she had neutropenia. During follow-up, with supportive treatment, liver enzymes and absolute neutrophil count returned to normal. No clinical or biochemical evidence of exocrine pancreas insufficiency was observed.

**Figure 1:**
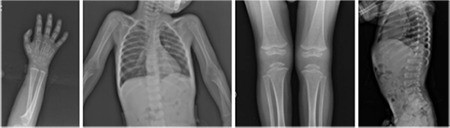
X-ray images show a generalized osteopenia, with epiphyseal dysplasia in the proximal tibia, distal femur, and proximal phalanges, additionally, tubulation deformities in the carpal bones and phalanges

At a routine visit at the age of 3 years and 5 months, her height was 90.9 cm (-1.53 standard deviation score) and weight was 11.3 kg (-2.45 standard deviation score). Her HbA1c was 8.69% while taking insulin detemir once a day and insulin lispro three times a day, with a total daily dose of insulin of 1.2 UI/kg. An X-ray survey showed osteopenia, generalized (proximal tibia, distal femur, and proximal phalanges) epiphyseal dysplasia, and tubulation deformities in the carpal bones and phalanges, but there were no abnormal findings in vertebral and pelvic bones (Figure 1).

A clinical diagnosis of WRS was corrected by the identification of a novel homozygous nonsense mutation (p.Q333) in exon 5 of the EIF2AK3 gene. [University of Exeter Medical School (United Kingdom) with funding from the Wellcome Trust to Professors Andrew Hattersley and Sian Ellard].

**Figure 2:**
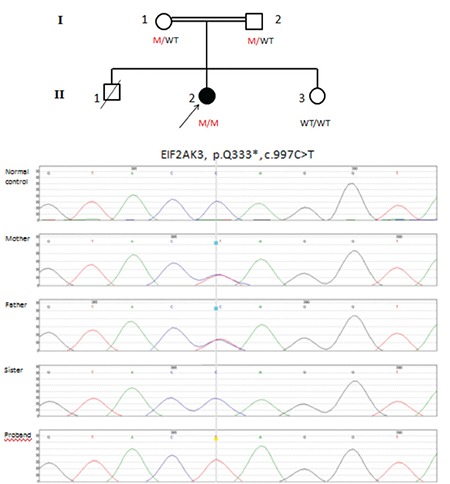
Electropherogram images for the mutation in the EIF2AK3 gene and pedigree of the family members

We name all our EIF2AK3 mutations according to the sequence reference AF110146.1. Patient’s parents were heterozygous for this mutation (Figure 2).

Hepatic dysfunction is a typical feature of this syndrome presenting with hepatomegaly, elevated hepatic enzymes, and recurrent acute liver failure (4). Our patient had one temporary acute hepatic attack, and it has not recurred. Although developmental delay has been reported in this syndrome, our patient’s development was normal.

The skeletal abnormalities of WRS are stated as progressive osteoporosis, osteopenia, and epiphyseal dysplasia (5). In our patient, osteopenia and generalized epiphyseal dysplasia were present.

In summary, the diagnosis of WRS should be considered in a neonatal or early infantile case of insulin-dependent diabetes mellitus with any of the accompanying features such as skeletal dysplasia, acute hepatic and/or renal failure, and neutropenia.

## Ethics

Peer-review: Externally peer-reviewed.

## References

[1] Wolcott CD, Rallison ML (1972). Infancy-onset diabetes mellitus and multiple epiphyseal dysplasia. J Pediatr.

[2] Mihci E, Turkkahraman D, Ellard S, Akcurin S, Bircan I (2012). Wolcott-Rallison syndrome due to a novel mutation (R491X) in EIF2AK3 gene. J Clin Res Pediatr Endocrinol.

[3] Ozbek MN, Senee V, Aydemir S, Kotan LD, Mungan NO, Yuksel B, Julier C, Topaloglu AK (2010). Wolcott-Rallison syndrome due to the same mutation (W522X) in EIF2AK3 in two unrelated families and review of the literature. Pediatr Diabetes.

[4] Julier C, Nicolino M (2010). Wolcott-Rallison syndrome. Orphanet J Rare Dis.

[5] Juneja A, Sultan A, Bhatnagar S (2012). Wolcott-Rallison syndrome. J Indian Soc Pedod Prev Dent.

